# A Case of Pseudo-pneumothorax with Complications

**DOI:** 10.7759/cureus.3263

**Published:** 2018-09-06

**Authors:** Azfar K Niazi, Paul Minko, Colin J Nahrstedt, Adam R Morris, Partha J Saha, Kavita Elliott, Tamer Ghaly, Sabry Ayad

**Affiliations:** 1 Outcomes Research, Cleveland Clinic, Cleveland, USA; 2 Anesthesia Institute, Cleveland Clinic Foundation - Fairview Hospital, Cleveland, USA; 3 Medical School, Ohio University Heritage College of Osteopathic Medicine, Athens, USA; 4 Medical School, Ohio University Heritage, Athens, USA; 5 Anesthesiology, Cleveland Clinic Fairview Hospital, Cleveland, USA

**Keywords:** pseudo-pneumothorax, pneumothorax, tube thoracostomy, skin fold

## Abstract

Pseudo-pneumothorax occurs after inappropriately diagnosing a pneumothorax based on a chest X-ray. This can be attributed to skin folds, bed sheets, previous pneumothorax, heating blankets, clothes, and other circumstances that may mimic the radiographic findings of a pneumothorax. We present a case where a patient underwent a tube thoracostomy due to the diagnosis of a pneumothorax that was not, in fact, present. The unnecessary intervention was complicated by hemoptysis and cardiac arrest.

## Introduction

Pseudo-pneumothorax, or "skin fold pneumothorax," is a finding on a plain chest radiograph that gives the false appearance of a pneumothorax. It can be caused by a number of conditions, including skin folds, bed sheet folds, an elevated hemidiaphragm, or a pleural cyst [1-4]. It is usually seen in patients with loose skin and can be reproduced when putting the film cassette behind the patient in sitting or supine position [1,5-6]. It can be differentiated from an actual pneumothorax by the presence of broncho-pulmonary markings beyond the false lung border [7]. The true incidence of pseudo-pneumothorax leading to intervention is not known. It is important to differentiate between a pneumothorax and a pseudo-pneumothorax because the latter is often benign and unnecessary intervention could precipitate serious complications.

## Case presentation

**Figure 1:**
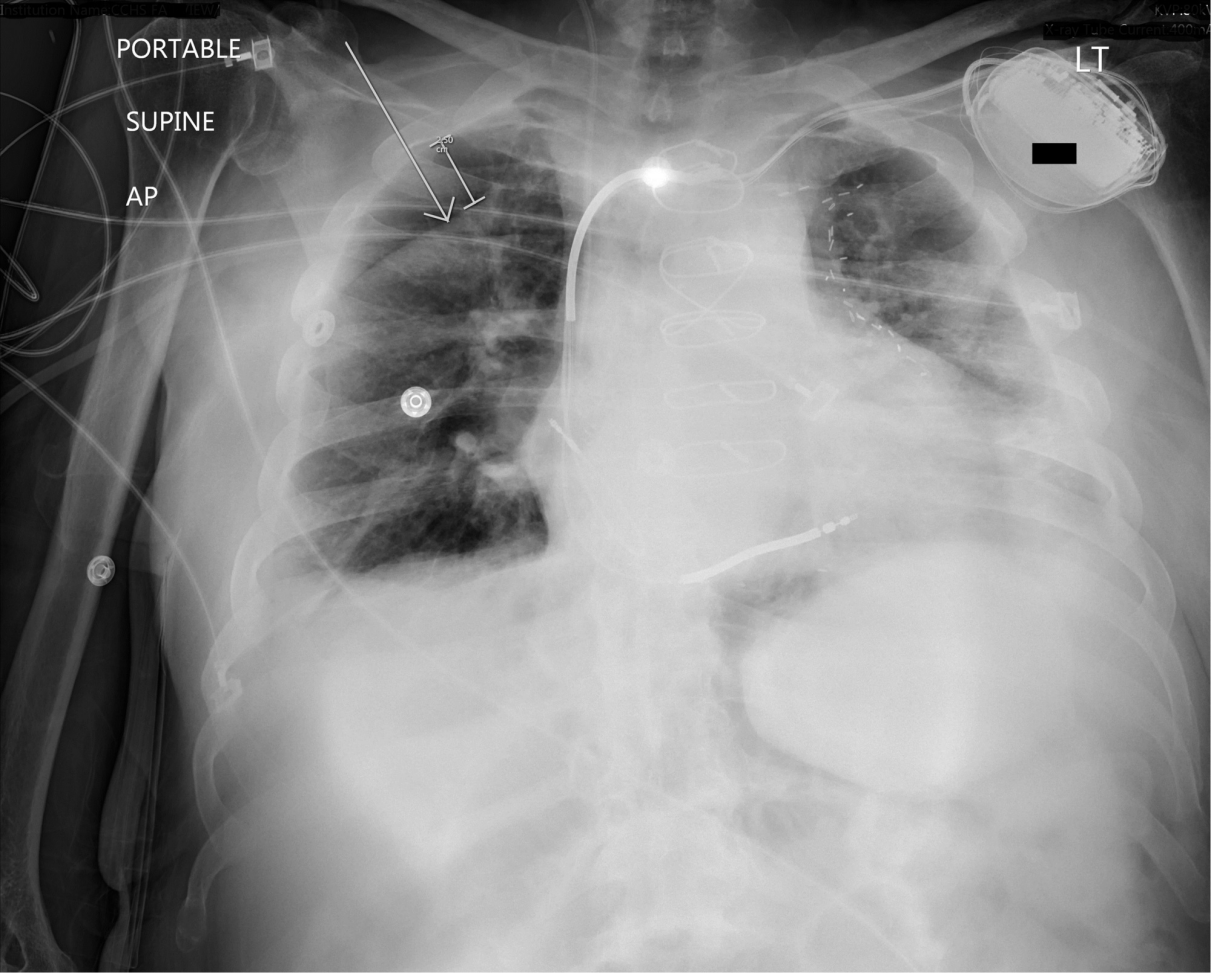
A skin fold can be seen giving a false appearance of pneumothorax in the top-right part of the lung AP: anteroposterior; LT: left

**Figure 2:**
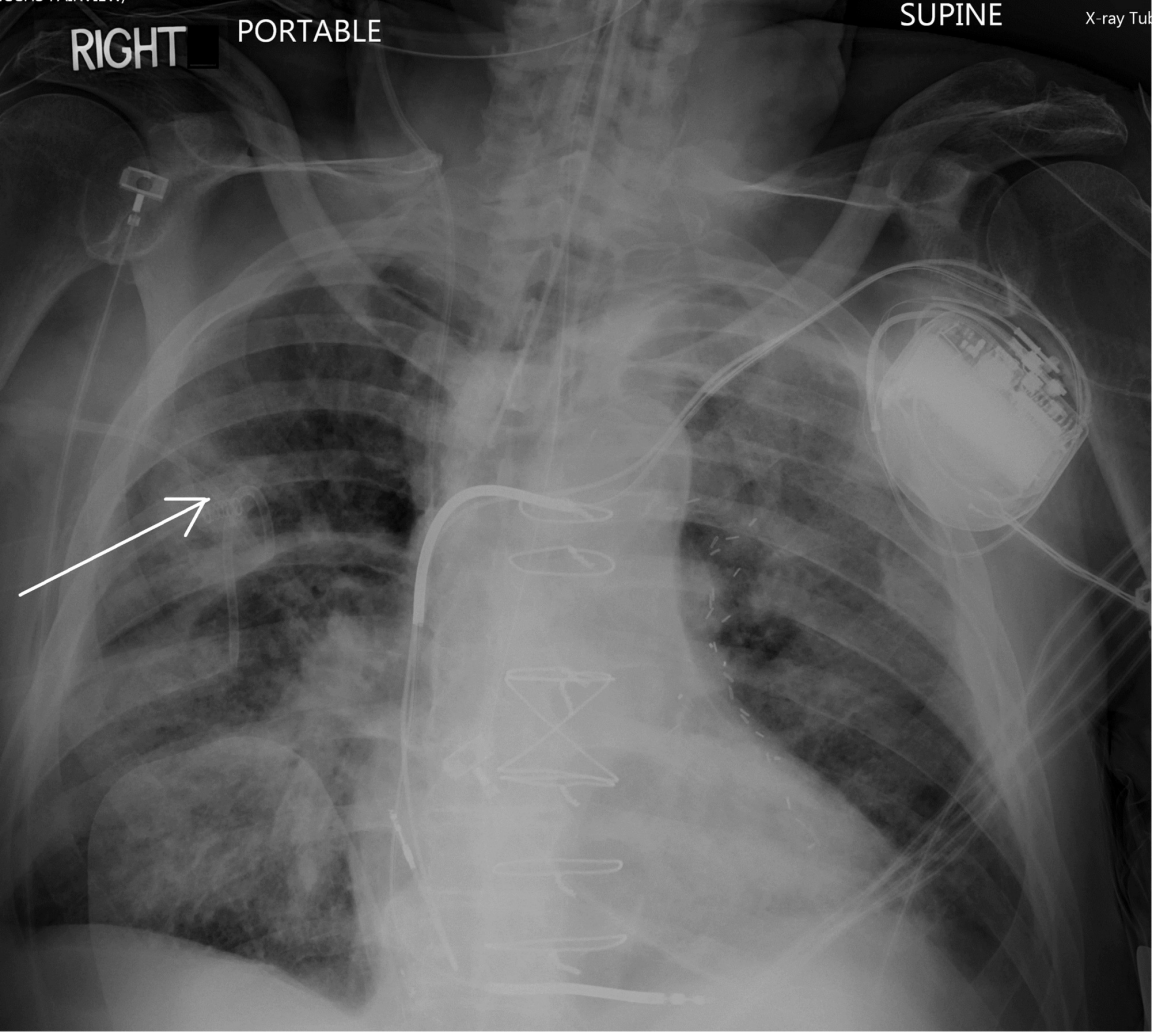
The arrow shows opacities representing intra-parenchymal/interstitial and intra-alveolar hemorrhage

**Figure 3:**
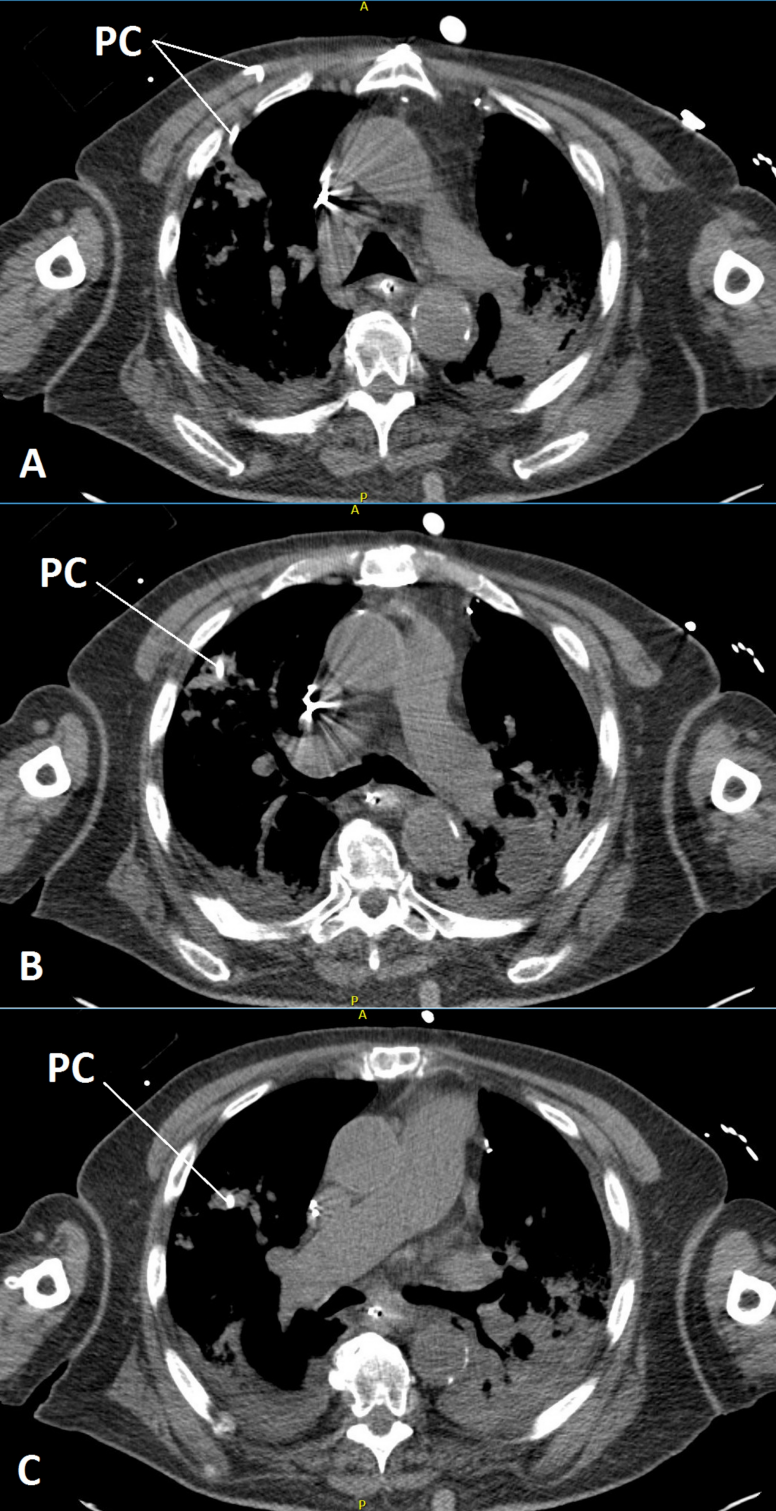
Series of computed tomography scanning showing a pigtail catheter going into the lung parenchyma and the surrounding hemorrhage/consolidation Three slices of computed tomography scans shown by A, B, and C (labeled in the lower-left corner of each scan). Small capital A and P on the top and bottom of each scan: A: anterior; P: posterior PC: pigtail catheter

A 75-year-old diabetic and hypertensive male with a complex past medical history—status post-coronary artery bypass surgery for coronary artery disease, ongoing long-term Coumadin therapy, and an automated, implantable cardioverter-defibrillator in place for chronic atrial fibrillation and low ejection fraction—presented with the classical signs and symptoms of appendicitis. The diagnosis was confirmed by a computed tomography (CT) scan of the abdomen. The patient was scheduled for a laparoscopic appendectomy and was admitted to the intensive care unit (ICU) because of hypotension secondary to septic shock. Laboratory results were significant for a white blood cell count of 20 k/µL, a serum creatinine level of 2.3 mg/dL, a blood urea nitrogen level of 50 mg/dL, and an international normalization ratio of 4.3. A right internal jugular vein central line was placed in a single attempt using ultrasound guidance for fluid resuscitation and vasopressor support. After adequate resuscitation, the patient was taken to an operating room and general anesthesia was induced. Prior to incision, a radiologist called into the operating room to report the presence of a right-sided pneumothorax (Figure 1). For safety, since the patient was on mechanical ventilation, a right-sided pigtail catheter was placed despite the absence of hemodynamic instability and a post-procedure x-ray was taken. Subsequently, the appendectomy was performed uneventfully. The patient was extubated and taken back to the ICU. Half an hour after extubation, the patient had several episodes of hemoptysis and oxygen saturation decreased to 70%, with complaints of shortness of breath and a muffled voice. The patient was emergently re-intubated, during which a cardiopulmonary arrest ensued. Cardiopulmonary resuscitation was performed according to advanced cardiac life support guidelines with a return of spontaneous circulation after 15 minutes. For the next hour, the patient was intubated and vital signs were recorded every 15 minutes and were reported as stable. After that, he was spontaneously opening his eyes, following commands, and was also able to move his legs and squeeze his right hand. A bronchoscopy was performed and showed the sloughing of the bronchial mucosa, causing a large amount of tissue obstructing the right main-stem bronchus and, to a lesser extent, the main carina and the endotracheal tube. The X-ray that led to the discovery of the pneumothorax was reviewed again with the radiology department. Upon review, the radiologist determined that a skin fold was misdiagnosed as a pneumothorax. A CT scan was performed, and it showed the catheter in the lung parenchyma coursing from the third right intercostal space through the right upper lobe and terminating adjacent to the minor fissure. An intra-parenchymal hemorrhage/consolidation along its course was also seen (Figure 2, Figures 3A-3C).

## Discussion

The identification of a pseudo-pneumothorax is of great clinical importance. Diagnosing it correctly can prevent an unnecessary tube thoracostomy and related complications. The CT scan revealed an intraparenchymal hemorrhage and lung consolidation, which is indicative of previous vessel injury during the thoracostomy procedure. This was severe enough to cause a severe decrease in oxygen saturation and cardiopulmonary arrest.

The presence of a pneumothorax was unlikely in the mentioned case because of various reasons. Firstly, the anesthesiologist used ultrasound guidance for the insertion of the central line, which lowers the risk of pneumothorax considerably [8]. Secondly, there was no clinical evidence to suggest the presence of a pneumothorax although the patient was anesthetized, which might have kept the clinicians from seeing any clinical signs.

A tube thoracostomy can cause certain complications. The case under discussion had supposed vascular injury leading to intraparenchymal hemorrhage. Kesieme et al. mentioned that major complications associated with tube thoracostomy include re-expansion pulmonary edema, subcutaneous emphysema, nerve damage to the phrenic and long thoracic nerves, heart perforation, pulmonary artery injury, subclavian artery occlusion, chest wall arteriovenous fistula, post-extubation fistula, infection, and esophageal perforation [9].

Radiological findings can often be skewed by a variety of factors. A case by Lee et al. showed a pneumothorax on a radiograph based on a curvilinear line at the right upper lung zone without definite lung markings peripherally. The CT scan ruled out a pneumothorax, leaving behind a skin fold as a possible cause [1]. Additionally, Kamath et al. published a case in which a bed sheet was found to be the cause of the artifact on a chest X-ray giving a false appearance of a pneumothorax [2]. In another case by Haq et al., a Bair warming blanket showed hyperlucency and lung volume loss on a chest X-ray with respiratory distress but equal breath sounds bilaterally. The chest tube placement was avoided by repeating the chest X-ray without the blanket, which resolved the pseudo-pneumothorax [10]. Moreover, similar scenarios have been observed in association with chest procedures. For example, Jacobs et al. wrote about a patient with suspected pneumothorax after the removal of the chest tube upon the resolution of the previous pneumothorax. A CT scan revealed that it was merely a cast of lung parenchyma near the chest tube placement site [11].

Imaging modalities, such as a CT scan and/or an ultrasound, along with a clinical assessment can reduce the risk of misdiagnosis. A CT scan is the gold standard test to diagnose pseudo-pneumothorax. However, cost and radiation exposure are important factors to consider, making a chest X-ray the most widely used screening option [12]. Ultrasonography is also a cost-effective, accurate, and readily available choice. Under the operation of radiologists, ultrasonography can be more accurate at diagnosing pneumothorax due to signs such as lung sliding, comet tail artifacts, the A-line sign, and lung point. That being said, Ding et al. advocate that the benefits of ultrasonography are obtained only when used by skilled operators and that their accuracy is directly proportional to it [13]. Per James Fisher, skin folds will oftentimes create a “curvilinear shadow over the lung parenchyma.” He also states that lung markings are present beyond these shadows, which should be enough evidence to rule out the presence of a pneumothorax. When looking at a potential pneumothorax on the periphery of the lung, these curvilinear lines can be seen as “broad, opaque bands” [7].

## Conclusions

A skin fold, along with a bed sheet fold, shirt fold, lung parenchyma shadow, and a warming blanket should be considered in the differential diagnoses of pneumothorax and should be carefully ruled out before making an intervention, especially when clinical suspicion for a pneumothorax is low.
